# Spatiotemporal cellular dynamics and molecular regulation of tooth root ontogeny

**DOI:** 10.1038/s41368-023-00258-9

**Published:** 2023-11-24

**Authors:** Pengcheng Rao, Junjun jing, Yi Fan, Chenchen Zhou

**Affiliations:** 1 State Key Laboratory of Oral Diseases & National Center for Stomatology & National Clinical Research Center for Oral Diseases & West China Hospital of Stomatology, Sichuan University, Chengdu, China; 2 State Key Laboratory of Oral Diseases & National Center for Stomatology & National Clinical Research Center for Oral Diseases & Department of Cariology and Endodontics, West China Hospital of Stomatology, Sichuan University, Chengdu, China; 3 State Key Laboratory of Oral Diseases & National Center for Stomatology & National Clinical Research Center for Oral Diseases & Department of Pediatric Dentistry, West China Hospital of Stomatology, Sichuan University, Chengdu, China

**Keywords:** Mesenchymal stem cells, Cell signalling

## Abstract

Tooth root development involves intricate spatiotemporal cellular dynamics and molecular regulation. The initiation of Hertwig’s epithelial root sheath (HERS) induces odontoblast differentiation and the subsequent radicular dentin deposition. Precisely controlled signaling pathways modulate the behaviors of HERS and the fates of dental mesenchymal stem cells (DMSCs). Disruptions in these pathways lead to defects in root development, such as shortened roots and furcation abnormalities. Advances in dental stem cells, biomaterials, and bioprinting show immense promise for bioengineered tooth root regeneration. However, replicating the developmental intricacies of odontogenesis has not been resolved in clinical treatment and remains a major challenge in this field. Ongoing research focusing on the mechanisms of root development, advanced biomaterials, and manufacturing techniques will enable next-generation biological root regeneration that restores the physiological structure and function of the tooth root. This review summarizes recent discoveries in the underlying mechanisms governing root ontogeny and discusses some recent key findings in developing of new biologically based dental therapies.

## Introduction

Dental integrity stands as an essential cornerstone of overall physiological well-being, given that teeth assume pivotal functions in processes such as mastication, digestion, phonation, and self-perception.^1–3^ The foundational essence of tooth anatomy is embodied within its root structure, which confers vital attributes of support, stability, and mechanosensory proficiency, thereby facilitating effective masticatory performance and upholding equilibrium within the alveolar bone milieu. Consequently, the loss of teeth instigates multifaceted detriments to both oral and systemic health, underscoring the exigency for swift and efficacious remedial interventions.^4^

Presently, osseointegrated dental implants emerge as the prevailing clinical benchmark for ameliorating tooth absence. However, it is noteworthy that certain limitations are attendant, encompassing elevated prerequisites for bone volume and an absence of sensory biofeedback mechanisms.^5–7^ In this context, nascent paradigms rooted in biological tooth regeneration have surfaced, leveraging the tenets of stem cell-based bioengineering.^8^ These innovative approaches portend outcomes that are more congruent with the innate physiology, durability, and bioresponsive dynamics of natural tooth structure, thereby heralding a promising and forward-looking therapeutic avenue.^9^

A pivotal prerequisite for advancing the therapeutic trajectory is the elucidation of ontogenetic processes that underpin the morphogenesis of tooth roots. This pursuit holds the promise of unveiling novel focal points for inciting intrinsic dental repair mechanisms and the orchestrated construction of tooth organ substitutes. The orchestration of tooth organogenesis ensues through a sequence of orchestrated, reciprocal molecular interplays between distinct specialized cell populations-the dental epithelial cohort and the cranial neural crest (CNC)-derived mesenchymal contingent.^10^ This intricate collaboration of molecular dialogs advances through discrete phases, beginning with the development of the tooth crown and terminating with the formation of the tooth root.

A strip of oral epithelium that has thickened marks the beginning of the creation of the crown, giving rise to the dental lamina.^11^ Following the accomplishment of crown development, the root morphogenesis commences, spearheaded by the inception of Hertwig’s epithelial root sheath (HERS). The orchestration of the signaling cascades in the HERS serves as the conductor directing odontoblast differentiation, ultimately leading to the deposition of radicular dentin. Concurrently, the dental follicle takes on the role of generating cementoblasts, which oversee the deposition of acellular and cellular cementum onto the nascent root surface, subsequent to the dissolution of HERS.

Throughout the trajectory of root ontogeny, the precise orchestration of spatiotemporally regulated signaling pathways assumes a nonpareil significance, serving as the compass directing HERS dynamics and the interplay between epithelial and mesenchymal compartments. Key protagonists in this narrative include signaling pathways entities, including Bone Morphogenetic Protein (BMP), Wnt, Transforming growth factor-β (TGF-β), Ectodysplasin A (EDA), and Sonic hedgehog (SHH), alongside a constellation of Mitogen‑activated protein kinase (MAPK) cascades.^12^ It is worth acknowledging that deviations from the norm within these signaling pathways choreographies underlie a panoply of root anomalies, spanning from abridged root dimensions to anomalies at the furcation junctures. Thoroughly deciphering the molecular mechanisms underlying the morphogenetic processes of tooth roots promises to unveil innovative perspectives for biotherapeutic interventions that activate intrinsic tooth repair and regeneration cascades.

## Spatiotemporal morphogenesis and cell source of dental root

Tooth development involves the lineage development of CNC cells, complex mutual interactions between dental epithelium and dental mesenchymal stem cells (DMSCs), and a precise sequence of morphological stages.^13,14^ The crown develops first, marked by the thickened oral epithelium proliferates and penetrates into the beneath CNC-derived mesenchyme and then give rise to the dental lamina structure.^15^ The dental lamina undergoes rapid cell proliferation, morphological expansion, and elongation, eventually developing into the enamel organ.^16,17^ Simultaneously, the CNC-derived mesenchyme condenses surrounding the dental lamina, serves as the major source of DMSCs. The dental lamina, together with the condensed mesenchyme around it, forms the tooth germ. The dental lamina develops into the enamel organ, and meanwhile the condensed mesenchyme diverges into the dental papilla and the dental follicle at the cap stage.^17,18^ The enamel organ produces enamel, while the dental papilla and the dental follicle subsequently form pulp-dentin complex and periodontal tissues, respectively.^19–21^

**Fig. 1:**
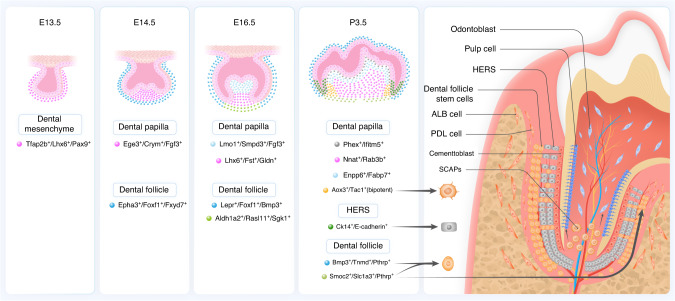
Lineage commitment and differentiation of dental stem cells involved in tooth root morphogenesis

The tooth germ experiences three stages in sequence during development: bud, cap, and bell stage. During mouse molar development, CNC-derived DMSCs separate into various cell populations and domains, which is a crucial phase in DMSCs differentiation and functional specialization (Fig. 1). At the bud stage, a homogenous population of Cranial neural crest-derived cells (CNCCs) marked by *Tfap2b*^*+*^*/Lhx6*^*+*^*/Pax9*^*+*^ in the condensed CNC-derived mesenchyme is identified as the progenitor cell population contributing to tooth formation. During the cap stage, the homogenous CNCCs segregate into dental papilla and dental follicle lineages, marked by specific genes like *Crym/Egr3/Fgf3* and *Epha3/Foxf1/Fxyd7* respectively.^9,17,18,21^ The dental papilla and follicle undergo further segregation at the bell stage. The dental papilla divides into distinct coronal and apical domains, marked by expression of *Lmo1/Fgf3/Smpd3* and *Lhx6/Fst/Gldn* respectively. Concurrently, the dental follicle divides into *Lepr/Foxf1/Bmp3* marked lateral domain and Aldh1a2/Rasl11a/Sgk1 marked apical domains. For instance, *Lepr*^*+*^ lateral follicle cells mainly form periodontal tissues. At the postnatal stage, dental papilla contains four distinct cell populations, including mature *Phex*^*+*^*/Ifitm5*^*+*^ odontoblasts, Enpp6/Fabp7^+^ coronal papilla, *Nnat*^*+*^*/Rab3b*^*+*^ middle papilla, and *Aox3*^*+*^*/Tac1*^*+*^ apical papilla cells. The latter was identified as bipotent progenitors for odontoblasts and pulp. In the follicle, *Pthrp*^*+*^*/Bmp3*^*+*^*/Tnmd*^*+*^ lateral and *Pthrp*^*+*^*/Smoc2*^*+*^*/Slc1a3*^*+*^ apical domains resemble those present at the bell stage and *Pthrp*^*+*^ dental follicle progenitors generate periodontal ligament (PDL) and cementum at the root-forming area and the furcation region.^17,22^ Particularly, a population of Slc1a3^+^ cells from the dental follicle’s apical area control cell lineages and aid in the formation of periodontal tissues at the root furcation. In addition, a range of other cells, such as glial cell derivatives, and pericytes can also serve as stem cell resources for dental mesenchyme.^15,23–25^ The perivascular niche contains *NG2*^*+*^, *Gli1*^*+*^, and *Acta2*^*+*^ cells that can differentiate into odontoblasts. The perineural niche is derived from Schwann cell precursors. Throughout the formation and regeneration following tooth damage, dental pulp cells and odontoblasts can be differentiated from *PLP1*^*+*^ and *Sox10*^*+*^ Schwann cells.^26^

Once the crown has formed during the late bell stage, the root development commences, marked by the formation of HERS.^27^ As the crown nears completion, the inner and outer enamel epithelium at the edge cervical loop continues to proliferate and elongate towards the future apical foramen from the cervical loop, which is located at the enamel organ’s cervical margin and is the region where the future crown and root of the tooth meet, also known as cementoenamel junction. This elongation gives rise to HERS, which is vital for the root development.^28^ *Gli1*^*+*^ cells, residing in HERS, apical dental papilla, and dental follicle, have been identified as progenitor cells in the root and play a crucial role during root development.^29^. Moreover, *Sox2*^*+*^ stem cells that resides in dental epithelial contribute to all epithelial cell lineages at the bell stage, while *Osx*^*+*^ progenitor cells that distributes in dental papilla and dental follicle, especially in the area adjacent to HERS, differentiate into odontoblasts, dental pulp cells, cementoblasts, and PDL cells.^22,30–32^ *Sox2*^*+*^ cells located at the cervical loop continue to exist in mouse incisors, while they disappear in mouse molars after birth.^33,34^ The cells from cervical loop and HERS, respectively, have their own different gene expression patterns. HERS cells have a relative higher level of BMP2 and BMP4, but a lower level the genes involved in enamel formation and mineralization, such as amelogenin (AMGN), ameloblastin (AMBN), osteopontin (OPN), bone sialoprotein (BSP), Osteocalcin (OCN) and dentin sialophosphoprotein (DSPP).^35^ HERS cells also express both *Cytokeratin 14 (Ck14)* and *Epithelial cadherin (E-cadherin)*, while detached cells from HERS are only *Ck14* positive with reduced *E-cadherin* expression.^36,37^

At the initial phase of HERS development, the ends of the double-layered enamel epithelium of HERS curve toward the dental papilla, forming an epithelial diaphragm structure, histologically encapsulating the dental papilla and surrounded by the dental follicle. This diaphragm has a determinantal role in root morphology, including shape, number, and length of the root.^38^ If the entire epithelial diaphragm boundary develops equally and ultimately merges, a normal single root will be formed. In the development of multirooted teeth, when the root is going to divide, several tongue-shaped epithelial diaphragms formed from HERS protrude toward each other and subsequently fuse to form continuous bridges, which will be the root furcation. The dental papilla is then divided into equivalent parts, ultimately resulting in the formation of equivalent roots.^39^

When the outermost mesenchymal cells of the apical papilla establish connections with the innermost cells of the HERS, they are induced to differentiate into odontoblasts and gradually retreat to the center of the dental papilla, secreting radicular dentin.^15,40,41^ As dentin formation progresses, the dental papilla becomes confined to a smaller space and eventually forms the dental pulp. Once the very first coat of radicular dentin is formed and the tooth root begins to elongate, HERS loses its integrity but remains a network of connections during root development through localized apoptosis or epithelial-to-mesenchymal transition (EMT), and the remaining HERS cells in the PDL are called ‘epithelial rests of Malassez (ERM)’, which contribute to cementum regeneration.^15,42–44^

Following the disintegration of the HERS, the lateral mesenchymal cells of the dental follicle acquire interaction with the radicular dentin and then differentiate into cementoblasts, forming acellular cementum and cellular cementum.^40,45^ In the region of the cervical root, cementoblasts first deposit cementum on the dentin and withdraw from the progressing mineralization front so that they do not incorporate themselves into the cementum, termed acellular cementum, also known as primary cementum. In more apical and inter-radicular areas, unlike acellular cementum formation, cellular cementum, also known as secondary cementum, is secreted by cementoblasts when the tooth reaches the occlusal plane, mineralizing rapidly and occasionally embedding some cementoblasts as cementocytes.^46–49^ The dental follicle also contributes osteoblasts and periodontal ligament cells to form the PDL and alveolar bone.^50^ In addition to dental follicle resources, some researchers have suggested that cementoblasts and PDL fibroblasts can also be derived from HERS cells through EMT and can be directly engaged in the deposition of cementum and PDL fibers during the development of periodontal tissues.^35,51^

The HERS serves as a critical regulator in the proper formation of the tooth root. One primary role of it in tooth root formation is to induce odontoblast differentiation and subsequent dentinogenesis.^42^ HERS development seems to be an especially solid procedure at the shift from the crown to the root morphological formation, whereas apical HERS development related to root elongation and the establishment of the root furcation appears to be more vulnerable to a variety of intrinsic as well as extrinsic negative effects.^52,53^. Developmental abnormalities in HERS can cause defects in tooth root formation.

When the continuity of HERS is compromised, stem cells from apical papilla (SCAPs) failed to differentiate into odontoblasts, leading to radicular dentin defects at that site and the formation of lateral accessory canals. Conversely, if HERS does not disintegrate after the first layer of dentin is formed and remains adhered to the dentin, it will block the engagement between dental follicle stem cells (DFSCs) and radicular dentin, preventing them from differentiating into cementoblasts and forming cementum. In some cases, delayed formation of the epithelial diaphragm in the multirooted teeth can result in taurodontism or single-rooted teeth. Following the completion of the bridges, future interradicular dentinogenesis and cementogenesis can be unaffected.^53^ Another phenomenon associated with failed HERS disintegration is the formation of enamel pearls. This occurs when HERS cells keep attached to the dentin and differentiate into ameloblasts, enamel pearls are formed.^54^

## Molecular regulation of HERS behaviors and epithelial-mesenchymal interactions

**Fig. 2:**
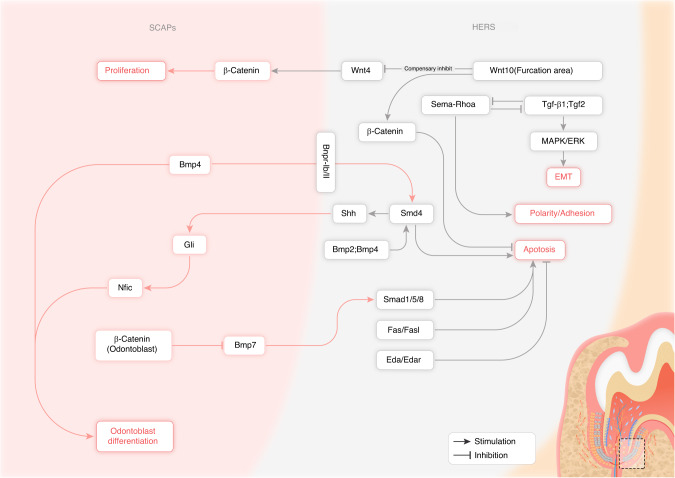
Signaling pathway cascades involved in HERS epithelial cells and the epithelial-mesenchymal interactions during tooth root development

Root formation is susceptible to various effects that can influence molecular processes. The underlying molecular mechanisms tightly coordinate morphogenic events, cytodifferentiation, mineralization, and maturation during tooth root development in a precise spatial and temporal way. Any disruptions of the involved regulatory mechanisms could lead to root malformations. The interaction and regulation among the signaling pathways and a range of molecular factors contribute to various processes during tooth root development (Fig. 2). Normal development of the tooth root requires a complex sequence and interaction between HERS and CNC-derived mesenchymal cell populations.^14^ Proper spatiotemporal coordination of these cell-cell interactions is mediated by several evolutionarily conserved signaling pathways.

The BMP signaling cascade, particularly BMP2 was crucial in determining root patterning and elongation.^55^ Simultaneous conditional deletion of *Bmp2* and *Bmp4* in dental epithelium resulted in persistent HERS with reduced apoptosis and downregulation of BMP/SMAD and MAPK (ERK, JNK) signaling pathways in HERS cells. The impaired BMP/SMAD signaling pathway further caused decreased expression of SHH in HERS and downregulated expression of GLI family zinc finger 1 (GLI1) in root mesenchyme cells, leading to downregulation of the downstream target gene Nuclear Factor I C (NIFC). Reduced NIFC ultimately resulted in defective differentiation and maturation of root odontoblasts, decreased Osterix (OSX) and DSPP expression, and impaired dentin formation, resulting in short root anomaly. *Dspp* transgenic expression partially alleviated the short root phenotype.^56^ Therefore, optimal spatiotemporal expression levels of epithelial BMP ligands were required to properly coordinate HERS dissolution and odontogenesis through intricate reciprocal epithelial-mesenchymal molecular interactions over the entire course of root development.^56^ However, one study suggested BMP4 ligand was secreted by surrounding DPSCs and bound to BMPR-IB and BMPR-II expressed on HERS epithelial cells. Organ culture studies employing developing mouse molars demonstrated that BMP4 derived from mesenchyme bound to BMPR-IB/II dimers on HERS cells, thereby providing negative feedback to inhibit further HERS elongation while simultaneously promoting odontoblast differentiation.^57^ Mice harboring an epithelial-specific deletion of *Smad4*, a pivotal intracellular BMP effector, exhibited arrested root developmental owing to failed HERS elongation and stratification, confirming SMAD4’s indispensable role.^58^ Specifical deletion of *Smad4* downregulated SHH expression within HERS and the surrounding inner enamel epithelium. Ectopic SHH expression in mutant mice partially rescued the root defects by restoring NFIC expression and normal dentin formation by newly differentiated odontoblasts.^59^ Epithelial SMAD4-SHH signaling pathway triggered mesenchymal NFIC expression to enable HERS morphogenesis and subsequent root formation. In contrast, conditional ablation of *Smad4* specifically in dental epithelium led to prolonged molar crown morphogenesis associated with maintained cervical loop structures harboring proliferative *Sox2*^*+*^ epithelial stem cells. Additional deletion of *Shh* in the epithelium in these compound mutant mice partially rescued this aberrant phenotype and eliminated *Sox2*^*+*^ epithelial stem cells. Hence, BMP-SMAD4 signaling pathway in the mesenchyme provided crucial negative feedback to restrict dental epithelial stem cell expansion during the later phase of root formation by suppressing the SHH-GLI1 signaling pathway in the epithelium.^33^

The canonical Wnt/β-catenin signaling pathway was also indispensable for proper differentiation of functional odontoblast lineage cells and root dentin formation. During root development, β-catenin was essential for preserving the proliferation, polarity, and adhesion of HERS cells. HERS-specific deletion or overexpression of *β-catenin* severely disrupted root morphogenesis and HERS dissociation. Studies suggested that suppressing β-catenin in HERS led to earlier disruption and thinner HERS, while stabilizing β-catenin in HERS prevented HERS dissociation after root dentin formation.^60^ Conditional deletion of *β-catenin* permitted molar tooth eruption accompanied by HERS elongation but completely abrogated root formation.^61^ Yet, odontoblast-specific deletion of *β-catenin* likewise caused premature pathological dissociation of HERS via upregulating BMP7, downregulating Noggin and Follistatin in odontoblasts, and accompanied by increased phosphorylation of SMAD1/5/8 in HERS cells.^14^ During molar furcation morphogenesis, canonical Wnt signaling pathway induced by epithelial WNT10A ligand critically patterned organized cell division and differentiation behaviors by modulating mesenchymal WNT4 expression and localized Wnt/β-catenin activity. In the furcation area, epithelial deletion of *Wnt10a* caused a decrease in epithelial cell proliferation but an increase in dental papilla mesenchymal cell proliferation, resulting in pathological taurodontism phenotype. *Wnt10a* deletion in dental epithelium causes compensatory upregulation of other Wnt ligands like WNT4, resulting in excessive β-catenin activation and overgrowth of dental papilla mesenchyme. Silencing of aberrant WNT4 partially rescued this abnormal furcation phenotype, validating that epithelial WNT10A precisely orchestrated dental epithelial and papilla mesenchymal cell proliferation by modulating the activation of mesenchymal WNT4 to guide root furcation morphogenesis.^62^

During the progression of tooth root maturation, HERS cells experienced EMT and relocated into the emerging periodontium. This EMT process could be induced in vitro by exogenous stimulation with either TGF-β1 or FGF2 growth factors, which activated MAPK/ERK intracellular signaling cascades.^63^ During EMT, HERS cells must precisely balance antagonistic Sema-RhoA and TGF-β signaling pathways, whereby the Sema-RhoA signaling pathway promoted the maintenance of epithelial polarity and adhesion while TGF-β signaling pathway drove the acquisition of mesenchymal attributes and enhanced migratory capacity.^36^ Furthermore, mesenchymal TGF-β signaling pathway indirectly help sustain the residual dental epithelial stem cell population by modulating Wnt signaling pathway activity in cervical loop through the induction of key ligands like WNT5a and FGF3/10^64^.

Moreover, DFSCs and cementoblasts could trigger apoptosis of both inner enamel epithelial-derived ameloblasts and HERS cells by upregulating FAS ligand (FASL) expression, which binds to and activates FAS-mediated extrinsic apoptotic signaling cascades.^65^ Eda-Edar signaling pathway represents another critical signaling pathway that directly participates in proper root formation and elongation. Both *Ectodysplasin A (Eda)* and *Ectodysplasin A receptor (Edar)* knockout mice exhibited substantially increased HERS proliferation and subsequent root developmental defects including shortened root length and taurodontism.^66^

In summary, intricate molecular signaling networks centered upon BMP, canonical Wnt/β-catenin, TGF-β, Fas/FasL, and Eda pathways precisely directed HERS behaviors and epithelial-mesenchymal interactions at distinct developmental stage of tooth root morphogenesis. Comprehensive elucidation of these intersecting regulatory mechanisms using advanced genetic models will uncover novel therapeutic targets to enhance endogenous tooth root repair and inform sophisticated bioengineering strategies for whole tooth regeneration.

## Molecular regulation of stem cells from apical papilla behaviors during tooth root development

**Fig. 3:**
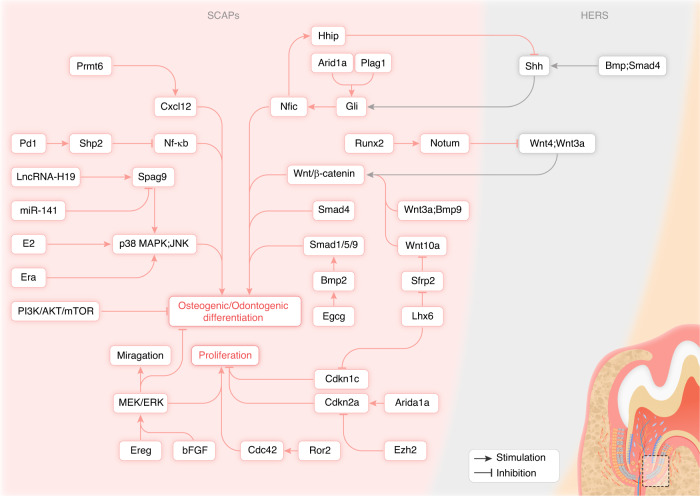
Molecular regulation of SCAPs for tooth root formation

Stem cells from apical papilla (SCAPs) express classic mesenchymal markers and have multi-differentiation potential.^67^ SCAPs have a considerably greater proliferation and mineralization capacity than dental pulp stem cells (DPSCs).^68,69^ During tooth development, a substantial number of SCAPs reside in the apical papilla. Through complex molecular signaling pathways, the apical papilla provides a niche regulating the organized differentiation of SCAPs. To promote further root elongation and dentin formation, SCAPs can differentiate into odontoblasts.^70^ The regulation of biological behaviors of SCAPs, such as differentiation, maturation, and mineralization, is tightly controlled by a cascade of signaling pathways, including TGF-β, WNT, and MAPK pathways (Fig. 3).^71^

TGF-β1 inhibited SCAPs proliferation and osteogenic differentiation by downregulating osteogenic genes like *Alp* and *Ocn* in a SMAD3-dependent manner. The TGF-β1’s inhibitory effect on SCAPs differentiation was enhanced by NFIC knockdown, whereas overexpression of NFIC antagonized these effects.^72^ Another study yielded the contrary results that TGF-β1 increased SCAPs proliferation and collagen production, which could be inhibited by blocking ALK5/SMAD2 and MEK/ERK pathways. Moreover, TGF-β1 had dual effects on regulation of Alkaline phosphatase (ALP) activity in SCAPs. For instance, low doses of TGF-β1 stimulated ALP activity which could be blocked by inhibiting ALK5/SMAD2 and MEK/ERK pathways, while higher doses inhibited ALP which could be rescued only by ALK5/Smad2 inhibition.^73^

The proper development of roots and deposition of dentin and cementum rely heavily on the precisely spatiotemporal management of the Wnt signaling pathway. In the mice with specifically deleted *β-catenin* in odontoblasts, it was observed that odontoblast differentiation was impeded and the expression of DSPP, OCN, and Type I collagen (COL I) in regions anticipated to form the root was significantly diminished. The loss of differentiated odontoblasts prevented root dentin formation, mineralization, and periodontal attachment, though the epithelial root sheath developed normally but failed to induce odontoblast differentiation in the absence of β-catenin signaling pathway.^74^ Low-dose Wnt agonist LiCl inhibited Glycogen synthase kinase 3 beta (GSK3β) and β-catenin degradation in nuclear, leading to β-catenin accumulation and stimulating cell cycle genes, thereby enhancing SCAPs proliferation and differentiation.^75^ WNT3a and BMP9 were reported to synergistically promote the osteogenic/odontogenic differentiation of SCAPs, which required functional β-catenin.^76^ Apurinic/Apyrimidinic (AP) endonuclease 1 (APE1) expression decreased during tooth development. APE1 inhibition promoted osteogenic differentiation of DPSCs via stimulating the canonical Wnt signaling pathway.^77^ Similar results were obtained that Secreted frizzled-related protein 2 (SFRP2) blocked Wnt signaling pathway activation by competing with Frizzled receptor for Wnt, increasing β-catenin phosphorylation and nuclear exclusion and thus downregulating Wnt target genes. *Sfrp2* overexpression enhanced while its knockdown inhibited osteogenic/odontogenic differentiation of SCAPs.^78,79^ LIM homeobox 6 (LHX6) coordinated Wnt activity and SCAPs differentiation to ensure correct root patterning and furcation development. LHX6 maintained active canonical Wnt signaling by inhibiting SFRP2 which physically interacted with WNT10A and suppressed WNT10A-induced odontoblast differentiation in the furcation area, resulting in defective dentin bridge formation. Moreover, LHX6 negatively regulated Cyclin-dependent kinase inhibitor 1C (CDKN1C) to maintain SCAPs proliferation.^80^ One recent study unraveled the tissue-specific role and molecular mechanisms of the non-canonical Wnt receptor, Receptor tyrosine kinase like orphan receptor 2 (ROR2), during molar root development. ROR2 was highly expressed in root-forming apical dental mesenchymal cells. Conditional deletion of *Ror2* in *Gli*^*+*^ DMSCs impaired root elongation and furcation formation, inhibited dental mesenchymal cell proliferation, disrupted HERS invagination, and decreased odontoblast differentiation through epithelial-mesenchymal interactions, but without affecting crown morphogenesis. Mechanistically, ROR2 regulated dental mesenchymal cell proliferation and root patterning likely by modulating the activity of Cell division cycle 42 (CDC42).^81^ However, Wnt inhibition might be required for normal development, as β-catenin overexpression in dental mesenchyme resulted in upregulated SMAD4 in odontoblasts while downregulation of Ectonucleotide pyrophosphatase/phosphodiesterase 1 (ENPP1) in cementoblasts, causing hypomineralized dentin and excessive cementum deposition respectively.^82,83^ Deletion of *Runt-related transcription factor 2* (*Runx2*) in *Gli1*^*+*^ DMSCs resulted in impaired odontoblast differentiation, downregulation of NOTUM and upregulation of WNT/β-catenin signaling. RUNX2 directly upregulated the NOTUM expression, which was specifically expressed in pre-odontoblasts and inhibited HERS-secreted WNT ligands, WNT3a as well as WNT4, and thus regulated WNT/β-catenin signaling in the dental mesenchyme.^84^

At-rich interaction domain 1 A (ARID1A) was also expressed in *Gli1*^*+*^ DMSCs during root development. *Arid1a* deletion in *Gli1*^*+*^ DMSCs resulted in shortened roots and impaired dentin and periodontal tissues. ARID1A interacted with transcription co-Factor Pleomorphic adenoma gene-like 1 (PLAGL1) and directly regulated the transcription of GLI1, thereby controlling the switch from proliferation to differentiation.^85^ Enhancer of zeste 2 polycomb repressive complex 2 subunit (EZH2) and ARID1A antagonistically regulated the expression of Cyclin dependent kinase inhibitor 2 A (CDKN2A) to control cell proliferation in the root-forming region, thereby coordinating furcation development and determining molar root number. Loss of EZH2 led to upregulation of CDKN2A, resulting in reduced cell proliferation and defective furcation formation. In contrast, monoallelic deletion of *Arid1a* rescued the root defects caused by loss of *Ezh2*.^86^

Furthermore, growth factors played crucial roles in apical papilla development. SCAPs expressed all four fibroblast growth factor (FGF) receptors. Basic FGF (bFGF) can activate Extracellular signal regulated kinase (ERK) and Transforming growth factor beta-activated kinase 1 (TAK1) phosphorylation in SCAPs. The MEK/ERK inhibitor U0126 and the TAK1 inhibitor 5Z-7-oxozeaenol compromise bFGF-induced upregulation of Plasminogen Activator Inhibitor-1 (PAI-1), Urokinase-type Plasminogen Activator (uPA), Urokinase Plasminogen Activator Receptor (uPAR), and Tissue Inhibitor of MetalloProteinase-1 (TIMP-1) expression. They also partially reversed bFGF-mediated inhibition of OCN expression in SCAPs. This suggests bFGF may regulate SCAPs proliferation, migration, and matrix turnover while suppressing differentiation through MEK/ERK and TAK1 signaling pathways.^87^ One study showed bFGF enhances stemness in SCAPs and decreases osteogenic/dentinogenic differentiation and related gene expression. Meanwhile, bFGF pretreatment enhanced the subsequent osteogenic differentiation potential of SCAPs.^88^ Epiregulin (EREG), an epidermal growth factor family member, stimulated the MEK/ERK and JNK signaling cascades, resulting in increased SCAPs proliferation.^89^

Additionally, lncRNA H19 promoted SCAPs osteogenic/odontogenic differentiation by binding to miR-141 and upregulating Sperm associated antigen 9 (SPAG9), which activated MAPK pathway by regulating the phosphorylation of p38 and JNK.^90^ 17beta-estradiol (E2) enhanced SCAPs osteogenic/odontogenic differentiation without affecting proliferation, by upregulating ALP, DSPP, Dentin matrix acidic phosphoprotein 1 (DMP1), and matrix mineralization and activating JNK, p38 MAPK pathways and downstream nuclear factors like c-Jun and c-Fos.^91^ Estrogen receptor α (Erα) also enhanced SCAPs osteogenic/odontogenic differentiation via the same mechanism.^92^

In addition to the previously delineated signaling pathways orchestrating the regulatory landscape of SCAPs, an array of noteworthy molecular factors also contributes to its behavior. Researchers found HERS cells secrete SHH ligand, and it antagonized NFIC which activated Hedgehog-interacting protein (HHIP) (a Hh inhibitor) to ensure normal Hh signaling pathway activity pattern in apical papilla, promoting proper apical papilla growth and root formation.^29^ Intriguingly, Lysine-specific demethylase 1A (KDM1A) had different regulatory roles at distinct stages during osteogenic/dentinogenesis. Inhibiting KDM1A reduced the activity of early osteogenic/dentinogenic markers and mineralization in SCAPs, but increased the expression of middle and late stage osteogenic/dentinogenic genes like *Bsp*, *Dspp*, and *Dmp1*. KDM1A can bind to Procollagen-lysine,2-oxoglutarate 5-dioxygenase 2 (PLOD2) to form a protein complex. Inhibition of PLOD2 had similar effects as inhibition of KDM.^93^ Additionally, there was a study suggested inhibition of PI3K/Akt/mTOR signaling pathway enhanced osteogenic/dentinogenic differentiation and calcification of SCAPs and SCAPs spheroids.^94^ Discoidin domain receptor family member 2 (DDR2) expressed in dental papilla, odontoblasts, dental follicle, as well as PDL during tooth development. Mice lacking the *Ddr2* gene exhibited short tooth roots, abnormal root/crown ratios, PDL defects, and progressive alveolar bone loss with impaired differentiation of cultured dental pulp and PDL cells.^95^ Moreover, Epigallocatechin-3-gallate (EGCG) facilitated osteogenic/odontogenic differentiation and mineralization of SCAPs by activating BMP2 expression and SMAD1/5/9 phosphorylation.^96^ Furthermore, the immune checkpoint molecule Programmed cell death 1 (PD1) that was expressed on SCAPs suppressed osteogenic differentiation by activating Src homology-2-containing protein tyrosine phosphatase 2 (SHP2) which inhibited its downstream NF-κB signaling pathway.^97^ Protein arginine methyltransferase 6 (PRMT6), a member of type-I enzymes, inhibited osteogenic/odontogenic differentiation of SCAPs by downregulating C-X-C motif chemokine ligand 12 (CXCL12) expression through increasing H3R2 methylation and forming a complex with Lamin A/C (LMNA) inhibiting its nuclear entry.^98^

Further insights into the regulatory network revealed that immune cells make up 83% of all tooth germ cells, demonstrating their regulatory responsibilities. They could directly modulate multiple processes during tooth development via secreted cytokines and acted on specific receptors, providing evidence for the novel roles of the immune system in tooth development.^99^

## Molecular regulation of dental follicle stem cell behaviors during root formation

**Fig. 4:**
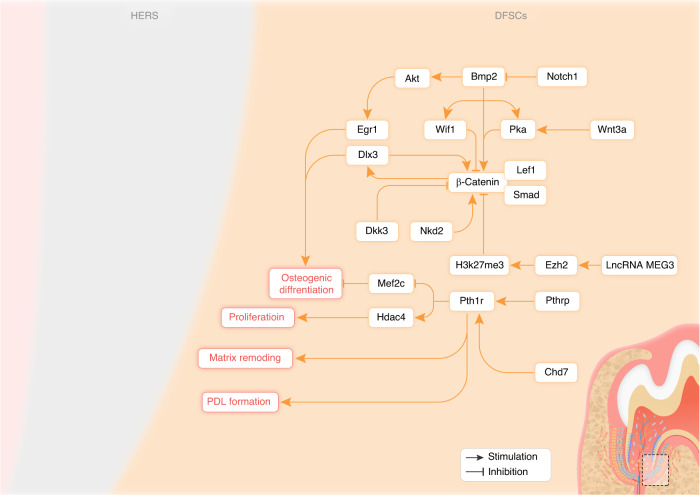
Signaling pathway regulation within DFSCs during tooth root development

DFSCs and DPSCs are both originated from dental mesenchyme, expressing classic markers of mesenchymal stem cells, including STRO-1, CD29, CD44, and CD146.^100,101^ Under specific induction conditions, when DFSCs and DPSCs were transplanted in vivo and simultaneously treated with dentin matrix, both cells could differentiate into odontoblast-like cells, express dentin-related markers and facilitate the dentin-pulp as well as the cementum-periodontium complex regeneration. However, DFSCs possess greater proliferation potential, osteogenic differentiation capacity, and mineralization ability compared to DPSCs. In contrast, DPSCs exhibit stronger angiogenic and dentinogenic potential.^102^ DFSCs are primarily responsible for the formation of cementum, PDL, and alveolar bone, which is governed by a number of signaling pathways (Fig. 4).

The precise balance of Wnt and BMP signaling pathways is critical for determining DFSCs fate. Canonical Wnt signaling pathway was required for DFSCs differentiation. Researchers co-cultured HERS cells and DFSCs of rat molars and observed increased mineralized nodule formation by DFSCs. The results suggested that HERS cells triggered the Wnt/β-catenin pathway in DFSCs through tight junctions with DFSCs, which in turn secreted extracellular matrix containing vesicles that promoted osteogenic differentiation of DFSCs and matrix mineralization.^103^ As a secreted Wnt inhibitor, Dickkopf-3 (DKK3), down-regulates the Wnt/β-catenin signaling pathway, leading to suppressed osteoblast differentiation of DFSCs. The expression of DKK3 was decreased during osteogenic differentiation of DFSCs. *Dkk3* knockdown enhanced the osteogenic potential of DFSCs, as evidenced by increased calcified nodules, and expression of osteogenic markers. *Dkk3*-deficient DFSCs also formed more osteoid matrix when implanted in vivo. These findings implied that DKK3 knockdown induced β-catenin accumulation in nuclear and activated canonical Wnt signaling pathway.^104^ Despite its role as a Wnt antagonist in other cell types, Naked cuticle homolog 2 (NKD2), an inhibitor of Wnt signaling pathway, enhanced the Wnt/β-catenin signaling pathway, thus promoted osteogenic differentiation of DFSCs.^105^

WNT3a was strongly expressed in HERS and crucial for the formation of dental roots by interacting with the BMP signaling pathway. BMP2 could activate the AKT signaling pathway and induce Early growth response factor 1 (EGR1) expression during osteogenic differentiation of DFSCs. Inhibition of AKT prevented EGR1 expression and osteogenic differentiation of DFSCs.^106^ An in vivo study showed BMP2 could induce cementoblast/osteoblast differentiation of DFSCs by intricate interaction between BMP and Wnt pathways to balance DFSCs fate. Exogenous WNT3a, a member of canonical Wnt signaling pathway, inhibited BMP2-induced differentiation, suggesting BMP-induced differentiation was suppressed by the Wnt/β-catenin signaling pathway. This was due to Wnt3a enhancing Lymphoid enhancer-binding factor 1 (LEF1), expression, which could inhibit RUNX2-dependent *Ocn* promoter activation. However, the β-catenin knockdown decreased BMP2-induced expression of RUNX2, ALP, and OCN, indicating endogenous Wnt/β-catenin signaling pathway was essential for BMP2 to induce DFSCs differentiation. BMP2 strongly induced Wnt inhibitor factor 1 (WIF1) expression, suggesting it initiated a negative feedback loop to control DFSCs maturation by inhibiting Wnt signaling pathway.^107^ Furthermore, the BMP2/PKA/β-catenin/DLX3 signaling pathway enhanced DFSCs osteogenic differentiation. BMP2 enhanced β-catenin transcriptional activity by activating PKA, which induced phosphorylation of β-catenin at Ser675. Activated β-catenin combined with LEF1 and SMAD4 to form a complex that promoted Distal-less homeobox 3 (DLX3) expression. Overexpression of *Dlx3* further activated the Wnt/β-catenin signaling pathway. In contrast, WNT3a suppressed BMP2-induced osteogenic differentiation of DFSCs by suppressing PKA activity.^108^ However, WNT3a was reported to initiate osteogenic commitment, promote the early cementoblast/osteoblast differentiation of DFSCs due to it activating the canonical Wnt signaling pathway and inducing the expression of osteogenic regulators. However, it was insufficient to drive terminal differentiation and mineralization which might be due to WNT3a inducing the expression of WNT5a to negatively regulate WNT3a-induced ALP and OSX expression.^109,110^ Similarly, a study revealed that WNT3a protein administration caused OSX overexpression, which could be blocked by p38 MAPK inhibitors but not by ERK or JNK inhibitors, indicating p38 MAPK activation was indispensable for WNT3a-mediated OSX induction. P38 MAPK activation was not affected by Dickkopf-1 (DKK1), suggesting WNT3a activated p38 MAPK independently of the Wnt/β-catenin pathway. Additional studies demonstrated inhibition of p38 MAPK did not impact WNT3a-induced phosphorylation of GSK3β or the levels and nuclear localization of β-catenin. However, p38 MAPK inhibition impaired Wnt3a-stimulated β-catenin transcriptional activity.^111^ Expression of Secreted frizzled-related protein 1 (SFRP1), a Wnt signaling pathway inhibitor, was significantly more prevalent in PDL cells than in dental follicle, cementoblasts, and alveolar bone cells. Inhibiting SFRP1 raised the active Trimethylation of histone H3 lysine 4 (H3K4me3) histone marked on the promoters of the *Runx2* and *Sp7* genes, boosting their expression and stabilizing β-catenin levels in DFSCs-derived PDL cells, enhancing β-catenin transcriptional activity.^112^ A study found that Long non-coding RNA maternally expressed gene 3 (lncRNA MEG3) combined with EZH2 inhibited the osteogenic differentiation of human DFSCs via blocking the Wnt signaling pathway. EZH2 was a histone methyltransferase that mediated Trimethylation at lysine 27 of histone H3 (H3K27me3). During osteogenic differentiation, EZH2 and H3K27me3 levels decreased while Wnt ligands and β-catenin increased in human DFSCs. Knockdown of lncRNA MEG3 or EZH2 promoted osteogenesis and Wnt pathway gene expression in human DFSCs. Chromatin immunoprecipitation showed EZH2 enrichment on Wnt gene promoters along with H3K27me3 accumulation, indicating epigenetic suppression. Inhibition of EZH2 reduced H3K27me3 occupancy on Wnt gene promoters, thus activating the Wnt/β-catenin signaling pathway to facilitate osteogenic differentiation.^113^

During cementogenesis, Parathyroid hormone-related peptide (PTHrP) was mainly produced by cells in the dental follicle. These *Pthrp*^*+*^ progenitor cells differentiated into PDL fibroblasts, acellular cementoblasts, as well as alveolar crypt bone osteoblasts.^21^ Parathyroid hormone receptor-1 (PTH1R) was a receptor for PTHrP and is expressed in *Osx*^*+*^, *Pthrp*^*+*^ and *Prx1*^*+*^ progenitor cells. PTHrP-PTH1R autocrine signaling pathway was required to maintain their physiological cell fates. In *Osx*^*+*^ dental mesenchymal progenitors, PTH1R deletion resulted in defects in cellular survival, proliferation, and aberrant differentiation, which caused shorter dental roots, PDL loss, and ankylosis. The PTH1R signaling pathway likely acts through Histone deacetylase 4 (HDAC4), as the PTH1R-deficient root condition was partially recapitulated by HDAC4 deletion.^22^ PTH1R also likely suppressed Myocyte enhancer factor 2 C (MEF2C) and bone matrix proteins to prevent precocious differentiation of *Pthrp*^*+*^ progenitors.^114^ *Prx1*^*+*^ cells were craniofacial mesenchymal progenitors expressing the transcription factor PRX1. When PTH1R was conditionally deleted in *Prx1*^*+*^ cells, it resulted in arrested incisor eruption and delayed molar eruption. PTH1R ablation caused decreased expression of osteogenic genes like *Runx2* and *Osx*, resulting in reduced alveolar bone formation. In vitro, PTH1R deletion suppressed osteogenic differentiation of DMSCs. The balance of bone remodeling was disrupted due to decreased bone formation when PTH1R was knocked out in *Prx1*^*+*^ cells. Reduced alveolar bone growth failed to provide sufficient eruptive force for tooth eruption. PTH1R deletion also led to aberrant PDL development with decreased Periostin expression. Therefore, the PTH1R signaling pathway in *Prx1*^*+*^ progenitors was indispensable for osteogenesis, bone remodeling, and PDL function during tooth eruption.^115^ Chromodomain-helicase-DNA binding protein 7 (CHD7) promoted osteogenic potential of human DFSCs by increasing PTH1R expression and enhancing PTH/PTH1R signaling pathway. PTH1R also was a key mediator of CHD7 regulation of osteogenic differentiation in human DFSCs as it was the direct transcriptional target of CHD7.^116^

Gene expression profiling reveals activation of BMP and TGF-β pathways during early osteogenic differentiation.^117^ In DFSCs, BMP9 transfection significantly elevated ALP activity, calcium deposition as well as osteogenic markers expression via activating non-canonical MAPK pathways, including increased p38 MAPK phosphorylation and decreased ERK1/2 phosphorylation.^118^. Interleukin-1alpha (IL-1α) bound to Interleukin-1 (IL-1) receptor on DFSCs and activated the downstream JNK and p38 MAPK pathways. Osteoclastogenesis was boosted and osteoblast differentiation was reduced when JNK and p38 MAPK were activated. These altered bone remodeling activities in dental follicle facilitate bone resorption around the erupting tooth and allow tooth eruption to proceed.^119^

RUNX2 played important roles in regulating osteogenic differentiation, matrix remodeling, and osteoclast induction in DFSCs. *Runx2* mutation in Cleidocranial dysplasia (CCD) patient DFSCs impaired matrix remodeling and osteoclast induction in patient DFSCs, which may cause delayed tooth eruption.^120,121^ But cellular senescence in the dental follicles was reduced in CCD patients compared to healthy controls. Isolated DFSCs from the patient showed increased proliferation, accelerated cell cycle, and downregulated senescence-associated genes and proteins. The patient’s DFSCs had decreased ERK phosphorylation, indicating a suppressed MAPK/ERK signaling pathway. Inhibition of ERK reduced senescence in control DFSCs, while ERK activation increased senescence in patient DFSCs. Therefore, the presence of the *Runx2* mutation in CCD prevents the cellular senescence process of DFSCs by blocking the ERK signaling pathway.^122^

Activated Notch1 signaling pathway was required in the osteogenic differentiation of DFSCs. Activation of the NOTCH1 inhibited osteogenic differentiation via BMP2/DLX3 pathway in DFSCs.^123^ Laminin inhibited early but promoted late osteogenic differentiation in human DFSCs through integrin-α2/β1 and FAK/ERK pathway.^14^ Laminin also induced mineralization and cementogenic gene expression while suppressing ALP activity in DFSCs.^124^ Transient receptor potential melastatin 4 (TRPM4) knockdown enhanced osteogenesis but inhibited adipogenesis of DFSCs by modulating calcium signaling pathway and related gene expression, suggesting a regulatory function for TRPM4 played in the differentiation of DFSCs.^125^

Heterogeneity was crucial for maintaining the properties of DFSCs. Subclones showed decreased osteogenic ability compared to heterogenous DFSCs. Selecting subclones disrupted the heterogeneity of DFSCs and altered their gene expression. The TGF-β signaling pathway had a significant impact on subclones’ ability to differentiate due to its cross-talk with the Hippo, Wnt, and stemness pathways.^126^

## Therapeutic implications and future directions

Strategies to modulate key developmental pathways could help stimulate innate dental repair mechanisms. For instance, local delivery of BMP ligands or agonists may promote HERS remodeling and odontoblast differentiation to treat root defects or stimulate reparative dentin formation. Modulating Wnt, Eda, PTH1R and FGF signalings could also direct dental stem cell behaviors. Further research should explore controlled, sustained delivery systems and optimize dosages for clinical translation.^127^

Tissue engineering approaches leveraging dental stem cells offer an attractive bio-inspired therapeutic avenue.^128^ However, recreating the complex microenvironmental dynamics of the native dental stem cell niche remains challenging.^129^ Smart biomaterial scaffolds that mimic key cues like signaling pathway molecules and architecture could guide dental stem cell organization into dental tissues.^130^ Emerging bioprinting techniques allow precise spatial control over the distribution of cells, signals, and materials to mimic native tissue architecture and function.^131^ Future efforts should uncover developmental mechanisms to inform scaffold and bioink design for precision engineering of the dentin-pulp complex.^132^ Combined innovations in dental stem cells, biomaterials, and bioprinting have immense potential to transform restorative dentistry through bioinspired tooth regeneration.

Patient-specific induced pluripotent stem cells (iPSCs) is a prospective cell source that can be applied in tooth regeneration and personalized dentistry.^133^ Recent studies have developed protocols to acquire iPSCs-derived dental epithelial and DMSCs using precisely orchestrated signaling pathways molecules like FGFs, BMPs, and WNTs.^134^ For example, Kim et al. generated dental epithelial-like stem cells from human ESCs and iPSCs through a stepwise procedure involving embryoid body formation, induction with retinoic acid, and keratinocyte serum-free medium.^135^ The iPSC-derived epithelial cells exhibited odontogenic capacity when recombined with mouse dental mesenchyme. Other research groups have guided iPSCs to form cranial neural crest-like cells, the odontogenic precursor tissue, which was further induced into functional odontoblast-like cells secreting dentin matrix upon FGF and BMP stimulation.^136,137^ Advanced 3D culture systems like peptide hydrogels have also been harnessed to enable the CNC-like cells to reproduce dental pulp-like tissue with embedded odontoblasts and vascularization in vivo.^136^ Overall, iPSCs offer an exciting platform for personalized biological tooth restoration, but further optimization of tooth organogenesis and maturation protocols will be critical for clinical translation.

The application of dental stem cell-derived stem cell aggregates has successfully emulated the intricate developmental processes of odontogenic mesenchymal cell condensation, culminating in the regeneration of damaged dental pulp in murine models and the restoration of pulp-like structures and functions reminiscent of normal dental pulp.^21,138–141^ Recent clinical trials have reinforced the translational promise of this approach in human subjects, promoting the regeneration of compromised dental pulp and periodontal tissues, thus enabling the repair of traumatized or avulsed teeth.^142^ This achievement in tooth tissue regeneration stands as a pivotal milestone in regenerative dentistry. To realize its full potential, it is imperative to optimize manufacturing processes, enhance regenerative efficacy, and ensure the stability of cell aggregates. In-depth explorations of interactions with host cells and tissues are essential to bolster biocompatibility. Further investigations into their performance in pathological microenvironments and the integration of biomaterials and bioactive molecules hold the potential for increased regenerative success.

In recent decades, enormous advancement has been made in dental tissue engineering, but the clinical translation of whole-tooth regeneration remains unsolved. Several key obstacles need to be addressed, including recapitulating the intricate spatiotemporal signaling pathway dynamics during odontogenesis, optimizing vascularization and innervation of bioengineered teeth, and ensuring proper integration with surrounding hard and soft tissues. Continued interdisciplinary research on innovative biomaterials, dental stem cells, growth factors, and advanced manufacturing techniques like bioprinting will be critical to unlock the full potential of biological tooth regeneration. Seamless integration of these emerging tools and technologies will usher in a new era of next-generation, bioinspired dental treatments that far surpass traditional prosthetic tooth replacements.

## Conclusion

Elucidating the intricate developmental mechanisms directing tooth root morphogenesis is critical to advance biological tooth restoration. Spatiotemporal orchestration of signaling pathways precisely regulates dental epithelial and mesenchymal stem cell behaviors during root formation. Thoroughly decoding these complex regulatory networks promises to uncover novel therapeutic targets to incite endogenous tooth repair mechanisms. Emerging tissue engineering that based on dental stem cells has enabled the engineering of biological tooth root replacements. However, recreating the native stem cell niche environments with proper biomaterials, growth factors, and architectural cues remains a key challenge for clinical translation. Advances in these areas that accurately recapitulate developmental dynamics will be instrumental to guide organized differentiation and functional integration of dental stem cells into bioengineered root structures. Moving forward, seamless integration of interdisciplinary innovations in developmental biology, stem cells, biomaterials, and advanced manufacturing techniques is imperative to realize the immense promise of transforming regenerative technologies into biologically based tooth root regeneration therapies that restore native physiological structure and function.

## References

[1] Cicalău, G. I. P. et al. Anti-inflammatory and antioxidant properties of carvacrol and magnolol, in periodontal disease and diabetes mellitus. *Molecules*10.3390/molecules26226899 (2021). 10.3390/molecules26226899 PMC8623889 34833990

[2] Friedlander L, Berdal A, Cormier-Daire V, Lyonnet S, Garcelon N (2023). Determinants of dental care use in patients with rare diseases: a qualitative exploration. BMC Oral. Health.

[3] Sahingur SE, Yeudall WA (2015). Chemokine function in periodontal disease and oral cavity cancer. Front. Immunol..

[4] Ostrovidov S (2023). Bioprinting and biomaterials for dental alveolar tissue regeneration. Front. Bioeng. Biotechnol..

[5] Safi IN, Hussein BMA, Al-Shammari AM (2022). Bio-hybrid dental implants prepared using stem cells with β-TCP-coated titanium and zirconia. J. Periodontal. Implant Sci..

[6] An, Y. Z., Heo, Y. K., Lee, J. S., Jung, U. W. & Choi, S. H. Dehydrothermally cross-linked collagen membrane with a bone graft improves bone regeneration in a rat calvarial defect model. *Materials*10.3390/ma10080927 (2017). 10.3390/ma10080927 PMC5578293 28796152

[7] Busenlechner D (2014). Long-term implant success at the Academy for Oral Implantology: 8-year follow-up and risk factor analysis. J. Periodontal. Implant Sci..

[8] Volponi AA, Pang Y, Sharpe PT (2010). Stem cell-based biological tooth repair and regeneration. Trends Cell Biol..

[9] Jamal, H. A. Tooth organ bioengineering: cell sources and innovative approaches. *Dent. J.*10.3390/dj4020018 (2016). 10.3390/dj4020018 PMC5851265 29563460

[10] Jussila M, Thesleff I (2012). Signaling networks regulating tooth organogenesis and regeneration, and the specification of dental mesenchymal and epithelial cell lineages. Cold Spring Harb. Perspect. Biol..

[11] Boy S, Crossley D, Steenkamp G (2016). Developmental structural tooth defects in dogs - experience from veterinary dental referral practice and review of the literature. Front. Vet. Sci..

[12] Liu Z, Lian W (2018). Molecular mechanisms of bone morphogenetic protein, Wnt, fibroblast growth factor and sonic hedgehog signaling pathways in tooth development. Chin. J. Tissue Eng. Res..

[13] Zhang W, Ju J, Gronowicz G (2010). Odontoblast-targeted Bcl-2 overexpression impairs dentin formation. J. Cell. Biochem..

[14] Zhang R (2015). Odontoblast β-catenin signaling regulates fenestration of mouse Hertwig’s epithelial root sheath. Sci. China Life Sci..

[15] Li J, Parada C, Chai Y (2017). Cellular and molecular mechanisms of tooth root development. Development.

[16] Kimura M (2022). The concurrent stimulation of Wnt and FGF8 signaling induce differentiation of dental mesenchymal cells into odontoblast-like cells. Med. Mol. Morphol..

[17] Jing J (2022). Spatiotemporal single-cell regulatory atlas reveals neural crest lineage diversification and cellular function during tooth morphogenesis. Nat. Commun..

[18] Yang C, Du XY, Luo W (2023). Clinical application prospects and transformation value of dental follicle stem cells in oral and neurological diseases. World J. Stem Cells.

[19] Omi M, Mishina Y (2022). Roles of osteoclasts in alveolar bone remodeling. Genesis.

[20] Liu, M., Goldman, G., MacDougall, M. & Chen, S. BMP Signaling pathway in dentin development and diseases. *Cells*10.3390/cells11142216 (2022). 10.3390/cells11142216 PMC9317121 35883659

[21] Sui BD (2023). Mesenchymal condensation in tooth development and regeneration: a focus on translational aspects of organogenesis. Physiol. Rev..

[22] Ono W, Sakagami N, Nishimori S, Ono N, Kronenberg HM (2016). Parathyroid hormone receptor signalling in osterix-expressing mesenchymal progenitors is essential for tooth root formation. Nat. Commun..

[23] Hermans F, Hemeryck L, Lambrichts I, Bronckaers A, Vankelecom H (2021). Intertwined signaling pathways governing tooth development: a give-and-take between canonical Wnt and Shh. Front. Cell Dev. Biol..

[24] Wakao S, Kuroda Y, Ogura F, Shigemoto T, Dezawa M (2012). Regenerative effects of mesenchymal stem cells: contribution of muse cells, a novel pluripotent stem cell type that resides in mesenchymal cells. Cells.

[25] Ibarretxe G (2012). Neural crest stem cells from dental tissues: a new hope for dental and neural regeneration. Stem Cells Int.

[26] Nagata M, Ono N, Ono W (2021). Unveiling diversity of stem cells in dental pulp and apical papilla using mouse genetic models: a literature review. Cell Tissue Res..

[27] Kumakami-Sakano M, Otsu K, Fujiwara N, Harada H (2014). Regulatory mechanisms of Hertwig׳s epithelial root sheath formation and anomaly correlated with root length. Exp. Cell Res..

[28] Bousnaki, M., Beketova, A. & Kontonasaki, E. A review of in vivo and clinical studies applying scaffolds and cell sheet technology for periodontal ligament regeneration. *Biomolecules*10.3390/biom12030435 (2022). 10.3390/biom12030435 PMC8945901 35327627

[29] Liu Y (2015). An Nfic-hedgehog signaling cascade regulates tooth root development. Development.

[30] Arai C (2017). Nephronectin plays critical roles in Sox2 expression and proliferation in dental epithelial stem cells via EGF-like repeat domains. Sci. Rep..

[31] Phattarataratip, E. et al. Expression of SOX2 and OCT4 in odontogenic cysts and tumors. *Head Face Med*. (2021). 10.1186/s13005-021-00283-1 PMC8278639 34261507

[32] Juuri E (2012). Sox2+ stem cells contribute to all epithelial lineages of the tooth via Sfrp5+ progenitors. Dev. Cell.

[33] Li J (2015). BMP-SHH signaling network controls epithelial stem cell fate via regulation of its niche in the developing tooth. Dev. Cell.

[34] Yu, T. & Klein, O. D. Molecular and cellular mechanisms of tooth development, homeostasis and repair. *Development*10.1242/dev.184754 (2020). 10.1242/dev.184754 PMC6983727 31980484

[35] Guo Y (2018). Comparative study on differentiation of cervical-loop cells and Hertwig’s epithelial root sheath cells under the induction of dental follicle cells in rat. Sci. Rep..

[36] Azumane M (2023). Semaphorin-RhoA signaling regulates HERS maintenance by acting against TGF-β-induced EMT. J. Periodontal. Res..

[37] Bi F (2023). Hertwig’s epithelial root sheath cells show potential for periodontal complex regeneration. J. Periodontol..

[38] Hosoya, A., Shalehin, N., Takebe, H., Shimo, T. & Irie, K. Sonic hedgehog signaling and tooth development. *Int. J. Mol. Sci*. 10.3390/ijms21051587 (2020). 10.3390/ijms21051587 PMC7084732 32111038

[39] Thanaruengrong P, Kulvitit S, Navachinda M, Charoenlarp P (2021). Prevalence of complex root canal morphology in the mandibular first and second premolars in Thai population: CBCT analysis. BMC Oral. Health.

[40] Guo H (2022). Development and regeneration of periodontal supporting tissues. Genesis.

[41] Jheon AH, Seidel K, Biehs B, Klein OD (2013). From molecules to mastication: the development and evolution of teeth. Wiley Interdiscip. Rev. Dev. Biol..

[42] Li X (2019). Development of immortalized Hertwig’s epithelial root sheath cell lines for cementum and dentin regeneration. Stem Cell Res Ther..

[43] Xiao L, Dudley AC (2017). Fine-tuning vascular fate during endothelial-mesenchymal transition. J. Pathol..

[44] Yamamoto T (2015). Hertwig’s epithelial root sheath fate during initial cellular cementogenesis in rat molars. Acta Histochem. Cytochem..

[45] Luan X, Ito Y, Diekwisch TG (2006). Evolution and development of Hertwig’s epithelial root sheath. Dev. Dyn..

[46] Yamamoto T, Hasegawa T, Yamamoto T, Hongo H, Amizuka N (2016). Histology of human cementum: Its structure, function, and development. Jpn Dent. Sci. Rev..

[47] Bosshardt DD, Stadlinger B, Terheyden H (2015). Cell-to-cell communication–periodontal regeneration. Clin. Oral. Implants Res..

[48] Foster BL (2012). Methods for studying tooth root cementum by light microscopy. Int. J. Oral. Sci..

[49] Pérez-Barbería FJ (2020). What do rates of deposition of dental cementum tell us? Functional and evolutionary hypotheses in red deer. PLoS ONE.

[50] Zhou T (2019). Dental follicle cells: roles in development and beyond. Stem Cells Int..

[51] Bertin TJC, Thivichon-Prince B, LeBlanc ARH, Caldwell MW, Viriot L (2018). Current perspectives on tooth implantation, attachment, and replacement in amniota. Front. Physiol..

[52] Al-Shahrani ZM, Balan U, Assiri KI, Al Qarni AMM (2020). Monoradicular primary mandibular first molar: a rare case in Aseer Province of Saudi Arabia. J. Oral. Maxillofac. Pathol..

[53] Luder HU (2015). Malformations of the tooth root in humans. Front. Physiol..

[54] Black, N. & Chai Y. Current understanding of the regulatory mechanism of tooth root development and future perspectives. *J. Calif. Dent. Assoc*. 10.1080/19424396.2023.2194560 (2023). 10.1080/19424396.2023.2194560 PMC10168653 37193003

[55] Yamamoto H (2004). Developmental properties of the Hertwig’s epithelial root sheath in mice. J. Dent. Res..

[56] Mu H (2021). Epithelial bone morphogenic protein 2 and 4 are indispensable for tooth development. Front. Physiol..

[57] Hosoya A, Kim JY, Cho SW, Jung HS (2008). BMP4 signaling regulates formation of Hertwig’s epithelial root sheath during tooth root development. Cell Tissue Res..

[58] Graf D, Malik Z, Hayano S, Mishina Y (2016). Common mechanisms in development and disease: BMP signaling in craniofacial development. Cytokine Growth Factor Rev..

[59] Huang X, Xu X, Bringas P, Hung YP, Chai Y (2010). Smad4-Shh-Nfic signaling cascade-mediated epithelial-mesenchymal interaction is crucial in regulating tooth root development. J. Bone Miner. Res..

[60] Yang S (2021). Cell dynamics in Hertwig’s epithelial root sheath are regulated by β-catenin activity during tooth root development. J. Cell Physiol..

[61] Kim TH (2013). β-catenin is required in odontoblasts for tooth root formation. J. Dent. Res..

[62] Yu M (2020). Epithelial Wnt10a is essential for tooth root furcation morphogenesis. J. Dent. Res..

[63] Chen J (2014). TGF-β1 and FGF2 stimulate the epithelial-mesenchymal transition of HERS cells through a MEK-dependent mechanism. J. Cell. Physiol..

[64] Yang G (2014). Mesenchymal TGF-β signaling orchestrates dental epithelial stem cell homeostasis through Wnt signaling. Stem Cells.

[65] Lee JH (2012). Dental follicle cells and cementoblasts induce apoptosis of ameloblast-lineage and Hertwig’s epithelial root sheath/epithelial rests of Malassez cells through the Fas-Fas ligand pathway. Eur. J. Oral. Sci..

[66] Fons Romero JM (2017). The impact of the eda pathway on tooth root development. J. Dent. Res..

[67] Amato, M., Santonocito, S., Viglianisi, G., Tatullo, M. & Isola, G. Impact of oral mesenchymal stem cells applications as a promising therapeutic target in the therapy of periodontal disease. *Int. J. Mol. Sci*. 10.3390/ijms232113419 (2022). 10.3390/ijms232113419 PMC9658889 36362206

[68] Zhang W (2014). Proliferation and odontogenic differentiation of BMP2 gene‑transfected stem cells from human tooth apical papilla: an in vitro study. Int. J. Mol. Med..

[69] Li G (2018). Local injection of allogeneic stem cells from apical papilla enhanced periodontal tissue regeneration in minipig model of periodontitis. Biomed. Res. Int..

[70] Driesen RB, Gervois P, Vangansewinkel T, Lambrichts I (2021). Unraveling the role of the apical papilla during dental root maturation. Front. Cell Dev. Biol..

[71] Diao, S. et al. Analysis of gene expression profiles between apical papilla tissues, stem cells from apical papilla and cell sheet to identify the key modulators in MSCs niche. *Cell Prolif.*10.1111/cpr.12337 (2017). 10.1111/cpr.12337 PMC6529102 28145066

[72] He W (2014). Regulatory interplay between NFIC and TGF-β1 in apical papilla-derived stem cells. J. Dent. Res..

[73] Chang HH (2015). Role of ALK5/Smad2/3 and MEK1/ERK signaling in transforming growth factor Beta 1-modulated growth, collagen turnover, and differentiation of stem cells from apical papilla of human tooth. J. Endod..

[74] Zhang R (2013). Disruption of Wnt/β-catenin signaling in odontoblasts and cementoblasts arrests tooth root development in postnatal mouse teeth. Int. J. Biol. Sci..

[75] Wang J, Liu B, Gu S, Liang J (2012). Effects of Wnt/β-catenin signalling on proliferation and differentiation of apical papilla stem cells. Cell Prolif..

[76] Zhang H (2015). Canonical Wnt signaling acts synergistically on BMP9-induced osteo/odontoblastic differentiation of stem cells of dental apical papilla (SCAPs). Biomaterials.

[77] Chen T (2015). Inhibition of Ape1 redox activity promotes odonto/osteogenic differentiation of dental papilla cells. Sci. Rep..

[78] Yu G (2016). Demethylation of SFRP2 by histone demethylase KDM2A regulated osteo-/dentinogenic differentiation of stem cells of the apical papilla. Cell Prolif..

[79] Jin L (2017). SFRP2 enhances the osteogenic differentiation of apical papilla stem cells by antagonizing the canonical WNT pathway. Cell Mol. Biol. Lett..

[80] He J (2021). Lhx6 regulates canonical Wnt signaling to control the fate of mesenchymal progenitor cells during mouse molar root patterning. PLoS Genet..

[81] Ma, Y. et al. Ror2-mediated non-canonical Wnt signaling regulates Cdc42 and cell proliferation during tooth root development. *Development*10.1242/dev.196360 (2021). 10.1242/dev.196360 PMC7847279 33323370

[82] Bae CH (2013). Excessive Wnt/β-catenin signaling disturbs tooth-root formation. J. Periodontal. Res..

[83] Li J (2011). SMAD4-mediated WNT signaling controls the fate of cranial neural crest cells during tooth morphogenesis. Development.

[84] Wen Q (2020). Runx2 regulates mouse tooth root development via activation of WNT inhibitor NOTUM. J. Bone Miner. Res..

[85] Du J (2021). Arid1a-Plagl1-Hh signaling is indispensable for differentiation-associated cell cycle arrest of tooth root progenitors. Cell Rep..

[86] Jing, J. et al. Antagonistic interaction between Ezh2 and Arid1a coordinates root patterning and development via Cdkn2a in mouse molars. *Elife*10.7554/eLife.46426 (2019). 10.7554/eLife.46426 PMC6602580 31259687

[87] Chang MC (2022). bFGF stimulated plasminogen activation factors, but inhibited alkaline phosphatase and SPARC in stem cells from apical Papilla: Involvement of MEK/ERK, TAK1 and p38 signaling. J. Adv. Res..

[88] Wu J (2012). Basic fibroblast growth factor enhances stemness of human stem cells from the apical papilla. J. Endod..

[89] Cao Y (2013). Epiregulin can promote proliferation of stem cells from the dental apical papilla via MEK/Erk and JNK signalling pathways. Cell Prolif..

[90] Li Z (2019). LncRNA H19 promotes the committed differentiation of stem cells from apical papilla via miR-141/SPAG9 pathway. Cell Death Dis..

[91] Li Y (2014). 17beta-estradiol promotes the odonto/osteogenic differentiation of stem cells from apical papilla via mitogen-activated protein kinase pathway. Stem Cell Res. Ther..

[92] Wang Y (2018). Oestrogen receptor α regulates the odonto/osteogenic differentiation of stem cells from apical papilla via ERK and JNK MAPK pathways. Cell Prolif..

[93] Wang L (2018). KDM1A regulated the osteo/dentinogenic differentiation process of the stem cells of the apical papilla via binding with PLOD2. Cell Prolif..

[94] Tanaka Y (2018). Suppression of AKT-mTOR signal pathway enhances osteogenic/dentinogenic capacity of stem cells from apical papilla. Stem Cell Res. Ther..

[95] Mohamed FF, Ge C, Binrayes A, Franceschi RT (2020). The role of discoidin domain receptor 2 in tooth development. J. Dent. Res..

[96] Liu, Z., Lin, Y., Fang, X., Yang, J. & Chen Z. Epigallocatechin-3-gallate promotes osteo-/odontogenic differentiation of stem cells from the apical papilla through activating the bmp-smad signaling pathway. *Molecules*10.3390/molecules26061580 (2021). 10.3390/molecules26061580 PMC8001198 33809391

[97] Li N (2022). PD-1 suppresses the osteogenic and odontogenic differentiation of stem cells from dental apical papilla via targeting SHP2/NF-κB axis. Stem Cells.

[98] Wang N (2022). PRMT6/LMNA/CXCL12 signaling pathway regulated the osteo/odontogenic differentiation ability in dental stem cells isolated from apical papilla. Cell Tissue Res..

[99] Shi Y (2021). A single-cell interactome of human tooth germ from growing third molar elucidates signaling networks regulating dental development. Cell Biosci..

[100] Diederic, A. et al. Influence of ascorbic acid as a growth and differentiation factor on dental stem cells used in regenerative endodontic therapies. *J. Clin. Med*. 10.3390/jcm12031196 (2023). 10.3390/jcm12031196 PMC9917775 36769844

[101] Tatullo M (2017). Potential use of human periapical cyst-mesenchymal stem cells (hPCy-MSCs) as a novel stem cell source for regenerative medicine applications. Front. Cell Dev. Biol..

[102] Guo L (2013). Comparison of odontogenic differentiation of human dental follicle cells and human dental papilla cells. PLoS ONE.

[103] Yang Y (2014). Hertwig’s epithelial root sheath cells regulate osteogenic differentiation of dental follicle cells through the Wnt pathway. Bone.

[104] Zhang X (2017). Dickkopf-related protein 3 negatively regulates the osteogenic differentiation of rat dental follicle cells. Mol. Med. Rep..

[105] Chen C, Zhang J, Ling J, Du Y, Hou Y (2018). Nkd2 promotes the differentiation of dental follicle stem/progenitor cells into osteoblasts. Int. J. Mol. Med.

[106] Viale-Bouroncle S, Klingelhöffer C, Ettl T, Morsczeck C (2015). The AKT signaling pathway sustains the osteogenic differentiation in human dental follicle cells. Mol. Cell. Biochem..

[107] Silvério KG (2012). Wnt/β-catenin pathway regulates bone morphogenetic protein (BMP2)-mediated differentiation of dental follicle cells. J. Periodontal. Res..

[108] Viale-Bouroncle S, Klingelhöffer C, Ettl T, Reichert TE, Morsczeck C (2015). A protein kinase A (PKA)/β-catenin pathway sustains the BMP2/DLX3-induced osteogenic differentiation in dental follicle cells (DFCs). Cell Signal..

[109] Sakisaka Y (2015). Wnt5a attenuates Wnt3a-induced alkaline phosphatase expression in dental follicle cells. Exp. Cell Res..

[110] Nemoto E (2016). Wnt3a signaling induces murine dental follicle cells to differentiate into cementoblastic/osteoblastic cells via an osterix-dependent pathway. J. Periodontal. Res..

[111] Sakisaka Y (2016). p38 MAP kinase is required for Wnt3a-mediated osterix expression independently of Wnt-LRP5/6-GSK3β signaling axis in dental follicle cells. Biochem. Biophys. Res. Commun..

[112] Gopinathan G, Foyle D, Luan X, Diekwisch TGH (2019). The Wnt antagonist SFRP1: a key regulator of periodontal mineral homeostasis. Stem Cells Dev..

[113] Deng L (2018). Down-regulated lncRNA MEG3 promotes osteogenic differentiation of human dental follicle stem cells by epigenetically regulating Wnt pathway. Biochem. Biophys. Res. Commun..

[114] Takahashi A (2019). Autocrine regulation of mesenchymal progenitor cell fates orchestrates tooth eruption. Proc. Natl Acad. Sci. USA.

[115] Cui C (2020). Role of PTH1R signaling in Prx1(+) mesenchymal progenitors during eruption. J. Dent. Res.

[116] Liu C, Li Q, Xiao Q, Gong P, Kang N (2020). CHD7 regulates osteogenic differentiation of human dental follicle cells via PTH1R signaling. Stem Cells Int..

[117] Aonuma H (2012). Characteristics and osteogenic differentiation of stem/progenitor cells in the human dental follicle analyzed by gene expression profiling. Cell Tissue Res..

[118] Li C (2012). Bone morphogenetic protein-9 induces osteogenic differentiation of rat dental follicle stem cells in P38 and ERK1/2 MAPK dependent manner. Int. J. Med. Sci..

[119] Meng M (2020). IL-1α regulates osteogenesis and osteoclastic activity of dental follicle cells through JNK and p38 MAPK pathways. Stem Cells Dev..

[120] Ge J (2015). Dental follicle cells participate in tooth eruption via the RUNX2-MiR-31-SATB2 loop. J. Dent. Res..

[121] Wang XZ (2016). RUNX2 mutation impairs 1α,25-dihydroxyvitamin d3 mediated osteoclastogenesis in dental follicle cells. Sci. Rep..

[122] Ji, L. et al. RUNX2 mutation inhibits the cellular senescence of dental follicle cells via ERK signalling pathway. *Oral Dis*. 10.1111/odi.14607 (2023). 10.1111/odi.14607 37154397

[123] Viale-Bouroncle S, Gosau M, Morsczeck C (2014). NOTCH1 signaling regulates the BMP2/DLX-3 directed osteogenic differentiation of dental follicle cells. Biochem. Biophys. Res. Commun..

[124] Viale-Bouroncle S, Gosau M, Morsczeck C (2014). Laminin regulates the osteogenic differentiation of dental follicle cells via integrin-α2/-β1 and the activation of the FAK/ERK signaling pathway. Cell Tissue Res..

[125] Nelson P (2013). Transient receptor potential melastatin 4 channel controls calcium signals and dental follicle stem cell differentiation. Stem Cells.

[126] Zhaosong M, Na F, Shuling G, Jiacheng L, Ran W (2021). Heterogeneity affects the differentiation potential of dental follicle stem cells through the TGF-β signaling pathway. Bioengineered.

[127] Gao W, Chen Y, Zhang Y, Zhang Q, Zhang L (2018). Nanoparticle-based local antimicrobial drug delivery. Adv. Drug Deliv. Rev..

[128] Siddiqui Z (2022). Cells and material-based strategies for regenerative endodontics. Bioact. Mater..

[129] Moussa DG, Aparicio C (2019). Present and future of tissue engineering scaffolds for dentin-pulp complex regeneration. J. Tissue Eng. Regen. Med..

[130] Yelick PC, Sharpe PT (2019). Tooth bioengineering and regenerative dentistry. J. Dent. Res..

[131] Monteiro N, Yelick PC (2017). Advances and perspectives in tooth tissue engineering. J. Tissue Eng. Regen. Med..

[132] Smith EE, Yelick PC (2019). Bioengineering tooth bud constructs using gelma hydrogel. Methods Mol. Biol..

[133] Otsu K (2014). Stem cell sources for tooth regeneration: current status and future prospects. Front. Physiol..

[134] Mai HN, Kim EJ, Jung HS (2021). Application of hiPSCs in tooth regeneration via cellular modulation. J. Oral. Biosci..

[135] Kim, G. H. et al. Differentiation and establishment of dental epithelial-like stem cells derived from human ESCs and iPSCs. *Int. J. Mol. Sci*. 10.3390/ijms21124384 (2020). 10.3390/ijms21124384 PMC7352334 32575634

[136] Kobayashi Y (2022). iPSC-derived cranial neural crest-like cells can replicate dental pulp tissue with the aid of angiogenic hydrogel. Bioact. Mater..

[137] Kim EJ (2021). Strategies for differentiation of hiPSCs into dental epithelial cell lineage. Cell Tissue Res..

[138] Wu M (2021). SHED aggregate exosomes shuttled miR-26a promote angiogenesis in pulp regeneration via TGF-β/SMAD2/3 signalling. Cell Prolif..

[139] Guo H (2021). Odontogenesis-related developmental microenvironment facilitates deciduous dental pulp stem cell aggregates to revitalize an avulsed tooth. Biomaterials.

[140] Chen H (2020). Regeneration of pulpo-dentinal-like complex by a group of unique multipotent CD24a(+) stem cells. Sci. Adv..

[141] Na S (2016). Regeneration of dental pulp/dentine complex with a three-dimensional and scaffold-free stem-cell sheet-derived pellet. J. Tissue Eng. Regen. Med.

[142] Xuan, K. et al. Deciduous autologous tooth stem cells regenerate dental pulp after implantation into injured teeth. *Sci. Transl. Med*. 10.1126/scitranslmed.aaf3227 (2018). 10.1126/scitranslmed.aaf3227 30135248

